# Operation Brain Trauma Therapy: An Exploratory Study of Levetiracetam Treatment Following Mild Traumatic Brain Injury in the Micro Pig

**DOI:** 10.3389/fneur.2020.586958

**Published:** 2021-01-13

**Authors:** Audrey Lafrenaye, Stefania Mondello, John Povlishock, Karen Gorse, Susan Walker, Ronald Hayes, Kevin Wang, Patrick M. Kochanek

**Affiliations:** ^1^Department of Anatomy and Neurobiology, Virginia Commonwealth University, Richmond, VA, United States; ^2^Department of Biomedical and Dental Sciences and Morphofunctional Imaging, University of Messina, Messina, Italy; ^3^Oasi Research Institute-IRCCS, Troina, Italy; ^4^Banyan Biomarkers, Inc., Alachua, FL, United States; ^5^Departments of Psychiatry & Neuroscience, Center for Neuroproteomics & Biomarkers Research, University of Florida, Gainesville, FL, United States; ^6^Department of Critical Care Medicine, University of Pittsburgh School of Medicine, Pittsburgh, PA, United States

**Keywords:** mild traumatic brain injury, levetiracetam, diffuse axonal injury, GFAP, micro pig, axonal regrowth, operation brain trauma therapy

## Abstract

Operation brain trauma therapy (OBTT) is a drug- and biomarker-screening consortium intended to improve the quality of preclinical studies and provide a rigorous framework to increase the translational potential of experimental traumatic brain injury (TBI) treatments. Levetiracetam (LEV) is an antiepileptic agent that was the fifth drug tested by OBTT in three independent rodent models of moderate to severe TBI. To date, LEV has been the most promising drug tested by OBTT and was therefore advanced to testing in the pig. Adult male micro pigs were subjected to a mild central fluid percussion brain injury followed by a post-injury intravenous infusion of either 170 mg/kg LEV or vehicle. Systemic physiology was assessed throughout the post-injury period. Serial serum samples were obtained pre-injury as well as at 1 min, 30 min, 1 h, 3 h, and 6 h post-injury for a detailed analysis of the astroglial biomarker glial fibrillary acidic protein (GFAP) and ubiquitin carboxy-terminal hydrolase L1. Tissue was collected 6 h following injury for histological assessment of diffuse axonal injury using antibodies against the amyloid precursor protein (APP). The animals showed significant increases in circulating GFAP levels from baseline to 6 h post-injury; however, LEV treatment was associated with greater GFAP increases compared to the vehicle. There were no differences in the numbers of APP+ axonal swellings within the pig thalamus with LEV treatment; however, significant alterations in the morphological properties of the APP+ axonal swellings, including reduced swelling area and increased swelling roundness, were observed. Additionally, expression of the neurite outgrowth marker, growth-associated protein 43, was reduced in axonal swellings following LEV treatment, suggesting potential effects on axonal outgrowth that warrant further investigation.

## Introduction

Traumatic brain injury (TBI) is a critical public health and socio-economic problem and, according to the World Health Organization, will soon become the third leading cause of death and disability worldwide (1–9). Despite many potential therapeutics that showed great promise in pre-clinical models, the list of negative or inconclusive clinical trials keeps growing, and the quest for effective therapeutic interventions for TBI continues. This unsuccessful clinical translation has triggered the rigorous standardization and refinement of TBI models and a call for the use of higher-order animals before moving to large-scale clinical trials (10–14). Accordingly, Operation Brain Trauma Therapy (OBTT), which is a pre-clinical therapy and biomarker screening consortium, aims to address barriers in the translation from preclinical to clinical studies in TBI. OBTT's approach incorporates heterogeneous types of brain injuries (three rat models mimicking different aspects of human TBI), sensitive outcome measures (histological, behavioral, and blood biomarkers), and rigorous standardized approaches to ensure reliability and reproducibility (15, 16). In addition, the workflow of the consortium dictates that the most effective therapies in OBTT's rodent studies move to testing in a large gyrencephalic micro pig model of TBI.

Levetiracetam (LEV), also known as Keppra, is an FDA-approved, second-generation antiepileptic agent that is commonly used in the clinic for the treatment of epilepsy and has been recommended for seizure prophylaxis following TBI (17). LEV is thought to reduce neuronal hyper-excitability through binding to the synaptic vesicle protein SV2a, which might also modulate the opening properties of the mitochondrial permeability transition pore (18, 19). Binding of LEV to SV2a has also been implicated in enhancing neurite outgrowth and increasing growth-associated protein 43 (GAP-43) expression (18). Administration of LEV following focal brain injury also resulted in an increase in growth-associated protein 43 expression, indicating a potential role for LEV in both the regulation of hyper-excitability and neurite regeneration following an injury (18, 20, 21).

LEV was the fifth drug tested by OBTT in three independent rodent models of moderate to severe TBI, namely, lateral fluid percussion injury (LFPI), cortical contusion injury (CCI), and penetrating ballistic-like brain injury (PBBI) (22). OBTT employs a scoring matrix to quantify and rank therapeutic efficacy, in which points are awarded for improved motor function (cylinder, grid walk, beam walk, rotarod), improved cognitive behavior (Morris Water Maze), reductions in glial fibrillary acidic protein (GFAP) and ubiquitin carboxy-terminal hydrolase L1 (UCH-L1) serum biomarker levels, and/or reduction of histopathology (lesion volume and hemispheric tissue loss) in each of the rodent models (15, 16, 23). Both doses of LEV (54 and 170 mg/kg) received a total of 10 out of a possible 66 points on the OBTT scoring matrix, making it the highest-scoring drug out of the 12 therapies tested to date (22, 23). The majority of these points were associated with cognitive improvement weeks following an injury using the Morris Water Maze test in the LFPI and CCI models (22). A reduction in hemispheric tissue loss was seen in the CCI model, and a reduction in serum GFAP levels at 24 h after an injury was seen with LEV *vs*. vehicle treatment in both CCI and PBBI models (22, 23). To date, LEV is the most promising drug tested by OBTT and therefore was advanced to testing in our well-established central fluid percussion injury (cFPI) micro pig model of mild diffuse TBI (24–26).

## Materials and Methods

### Animals

Experiments were conducted in accordance with the Virginia Commonwealth University institutional guidelines concerning the care and use of laboratory animals (Institutional Animal Care and Use Committee), which adhere to regulations including, but not limited to, those set forth in the “Guide for the Care and Use of Laboratory Animals: 8^th^ Edition” (National Research Council). Fifteen adult male Yucatan micro pigs, weighing 17–28 kg (~6 months of age), were used for this study. One animal was excluded from analysis based on our *a priori* exclusion criteria of sustaining gross focal brain damage. The animals were housed in pairs in environmentally controlled pens on a 12-h light–dark cycle, with free access to food and water. We selected a 6-h post-injury time point for these initial translational studies for the following reasons: (1) levels of GFAP are significantly elevated in the serum of clinical TBI patients by 5–8 h post-injury (27, 28) and (2) our pre-determined histological outcome for this model was DAI, which traditionally occurs acutely (within hours) post-injury and is consistently present in our micro pig model of cFPI at 6 h (24).

### Surgical Preparation and Injury Induction

The micro pigs were initially anesthetized with an intramuscular injection of 100 mg/ml xylazine (2.2 mg/kg; AnaSed Injection, Shenandoah, IA, USA) and 100 mg/ml telazol (2.0 mg/kg; tiletamine HCL and zolazepam HCL; Pfizer, New York, NY, USA), followed by an intravenous administration of sodium pentobarbital (60 mg/kg; Sigma-Aldrich, St. Louis, MO, USA). Once the absence of a corneal reflex was verified, the micro pig was intubated and ventilated with 1 to 2% isoflurane mixed in 100% oxygen throughout the experiment. Ophthalmic lubricant (Dechra, Overland Park, KS, USA) was applied to avoid damage or drying of the eye. Body temperature was monitored with a rectal thermometer and maintained at 37°C with a heating pad. Catheters were placed in the right femoral artery and vein for continuous monitoring of the mean arterial blood pressure (MABP), assessment of blood gases, and infusion of drug or vehicle treatment, as described below, or Lactated Ringer's solution (Hospira, Lake Forest, IL, USA) to maintain hydration. A midline incision was made from the supraorbital process to the nuchal crest, and a 14-mm-diameter circular craniotomy was trephined along the sagittal suture, positioning the center of the craniotomy 15 mm anterior to lambda (on the nuchal crest) and leaving the dura mater intact. A stainless steel custom-threaded hub (Custom Design and Fabrication, Richmond, VA, USA) was screwed into the craniotomy site to a depth of ~4 mm. Screws were then placed directly posterior and anterior-lateral to the craniotomy, and dental acrylic (methyl-methacrylate; Hygenic Corp., Akron, OH) was applied around the hub and screws to insure hub stability. Induction of the central fluid percussion injury (cFPI) was done as previously described (24, 26). Briefly, the anesthetized micro pigs were connected to a cFPI device retrofitted with an L-shaped stainless steel adaptor that allowed for a sealed connection to the injury hub. The micro pigs were then injured at a magnitude of 1.7 ± 0.2 atmospheres with a pressure pulse measured by a transducer affixed to the injury device and displayed on an oscilloscope (Tektronix, Beaverton, OR, USA). Immediately after injury induction, the animals were disconnected from the injury device, the screws and hub were removed from the bone, and the dental acrylic, hub, and screws were removed *en bloc*. This injury did not result in any breach of the dura mater. Gel foam was placed over the craniotomy/injury site to alleviate minute bone bleeding, and the scalp was sutured. The animals were maintained anesthetized for the duration of the 6-h post-injury monitoring period.

### Drug Administration

The animals were randomly assigned to two groups: injury + vehicle (normal saline) and injury + LEV. Clinical-grade LEV (100 mg/ml) was obtained from Caraco Pharmaceutical Laboratories (Detroit, MI) or X-Gen Pharmaceuticals, Inc. (Big Flats, NY). The micro pigs received either 100 ml of sterile normal saline (*n* = 7) or 170 mg/kg LEV (*n* = 7) dissolved in sterile physiologic saline to a final total volume of 100 ml. This was given beginning 15 min following cFPI *via* a slow intravenous infusion over a 45-min period. The dose of LEV was chosen based on OBTT's previous finding in our rodent studies (22).

### Systemic Physiological Assessment

Systemic physiological assessments were performed prior to injury and throughout the 6-h post-injury monitoring period. Heart rate, arterial blood pressure, rectal temperature, and hemoglobin oxygen saturation were monitored and recorded throughout the experiment *via* a Cardell® MAX-12HD (Sharn Veterinary, Inc., Chicago, IL, USA). The femoral artery was cannulated for continuous monitoring of MABP and for blood sampling to determine arterial oxygen tension (PaO_2_), arterial carbon dioxide tension (PaCO_2_), and pH values using a Stat Profile pHOx (NOVA Biomedical, Waltham, MA, USA). The resting PaCO_2_ level was maintained between 35 and 40 mmHg by adjusting the rate and/or tidal volume of the respirator. All animals maintained physiological homeostasis (i.e., 60 mmHg < MABP <130 mmHg, hemoglobin oxygen saturation >90%, 90 BPM < heart rate <140 BPM; Table 1).

**Table 1:** Injury parameters and systemic physiology of vehicle- and levetiracetam-treated micro pigs prior to and throughout the 6-h post-injury monitoring period.

	**Pre-traumatic brain injury (TBI)**		**Post-TBI**	
	**Vehicle**	**Levetiracetam**	**Vehicle**	**Levetiracetam**
Weight (kg)	23.04 ±2.69	21.60 ± 2.35		
Injury intensity (atm)	1.72 ±0.14	1.73 ± 0.11		
Injury duration (ms)	31.23 ±2.79	30.84 ± 1.61		
PaO_2_ (mmHg)	546.41 ± 63.59	537.38 ± 57.61	480.89 ± 72.00^*^	453.37 ± 79.87^*^
PaCO_2_ (mmHg)	40.41 ± 3.85	40.34 ± 6.40	37.72 ± 1.75	37.72 ± 1.30
pH	7.50 ± 0.02	7.49 ± 0.05	7.52 ± 0.04	7.50 ± 0.02
Hemoglobin O_2_ (%)	99.73 ± 0.16	99.87 ± 0.05	99.36 ± 0.54^*^	99.45 ± 0.27^*^
MABP (mmHg)	104.25 ± 13.64	104.48 ± 6.29	95.97 ± 13.56	107.21 ± 10.25
Heart rate (BPM)	128.98 ± 16.48	121.31 ± 10.79	115.10 ± 13.84	110.99 ± 5.09

BPM, beats per minute; MABP, mean arterial blood pressure.

Physiology data are given as mean ± standard deviation of the mean.

* Significant difference compared to pre-injury readings for the same group (p < 0.05).

#### Detection and Quantification of Serum Biomarker Levels

Serial arterial blood samples of 3 ml were obtained pre-cFPI (pre-craniotomy and post-craniotomy) as well as at 1 min, 30 min, 1 h, 3 h, and 6 h post-injury. Blood volume was replaced with an intravenous infusion of Lactated Ringer's solution (Hospira, Lake Forest, IL, USA) to maintain proper hydration. All blood samples were processed to obtain serum according to the OBTT manual of standard operating procedures and stored at −80°C prior to shipment to Banyan Biomarkers for analysis. Blood levels of GFAP and UCH-L1 were measured by enzyme-linked immunosorbent assay (ELISA) using proprietary anti-GFAP and anti-UCH-L1 antibodies (please see Mondello and associates as well as Shear and colleagues for a more detailed description of the ELISA and the biomarker-related methods used in these studies) (13, 29).

### Tissue Processing

At 6 h after, the cFPI micro pigs were administered with a euthanizing dose (3 ml) of euthasol euthanasia-III solution (Henry Schein, Dublin, OH; USA), transcardially perfused with 0.9% saline, followed by 4% paraformaldehyde/0.2% gluteraldehyde in Millonig's buffer (136 mM sodium phosphate monobasic/109 mM sodium hydroxide) for immunohistochemical analysis. After transcardial perfusion, the brains were removed and post-fixed in 4% paraformaldehyde/0.2% gluteraldehyde/Millonig's buffer for 36–48 h. Postfixed brains were blocked into 5-mm coronal segments throughout the rostral–caudal extent using a tissue slicer (Zivic Instruments, Pittsburgh, PA, USA). The thalamus was chosen as the region of interest for histological assessment based on the consistent involvement of the thalamic domain in TBI as well as our previous studies demonstrating thalamic involvement in our pig model of cFPI (24, 26, 30). Segments containing the thalamus were bisected at the midline, and the left side was analyzed. The 5-mm coronal segments containing the thalamus were coronally sectioned in 0.1 M phosphate buffer with a vibratome (Leica, Banockburn, IL, USA) at a thickness of 40 μm. Sections were collected serially in six-well plates (240 μm between sections in each well) and stored in Millonig's buffer at 4°C. For immunohistological quantification, the serially collected tissue was selected from a single well in the six-well plate. This well was determined using a random number generator, and four sections, representing the rostral–caudal axis contained within the selected well, were analyzed. All histological analyses were restricted to the thalamus using anatomical landmarks and were performed by an investigator blinded to the animal treatment groups (vehicle or LEV).

### Detection and Quantification of Injured Axonal Swellings

To visualize axonal transport issues indicative of axonal injury, immunohistochemistry targeting the normally expressed and anterogradely transported amyloid precursor protein (APP) was performed. In this procedure, four sections per animal were blocked and permeabilized in 10% normal goat serum and 1.5% triton, followed by overnight incubation with a primary rabbit antibody against the C-terminus of β-APP (1:700; Cat. #51-2700, Life Technologies, Carlsbad, CA, USA) at 4°C. Secondary antibody Alexa Fluor 568-conjugated goat anti-rabbit IgG (1:500; Cat. #A-11011, Life Technologies, Carlsbad, CA, USA) was then incubated, and tissue was mounted on slides using Vectashield hardset mounting medium with DAPI (Cat. #H-1500; Vector Laboratories, Burlingame, CA, USA). Tissue sections from all animals were processed concomitantly to obviate variability in staining intensity. Visualization of APP-labeled axonal swellings was performed using a Nikon Eclipse 800 microscope (Nikon, Tokyo, Japan) equipped with an Olympus DP71 camera (Olympus, Center Valley, PA, USA). Images (40 images from four sections per animal) were taken by a blinded investigator at × 10 magnification (0.72-mm^2^ field) in a systematically random fashion using DAPI to verify focus and restriction within the thalamic region of interest. Image acquisition settings were held constant for all animals. Analysis of the number of APP+ axonal swellings was performed using the particle analysis function in FIJI image analysis software (NIH, Bethesda, MD). The number of APP+ swellings per unit area was quantified for each image and averaged for each animal.

To evaluate the number of injured axons following vehicle or LEV treatment, the total number of APP+ axonal swellings within the thalamic region of four systematically random-sampled sections throughout the rostral–caudal extent of the pig brain was counted by two independent investigators blinded to the animal groups. Data are expressed as number of APP+ swellings per section.

To assess injured axonal swelling morphology, four fluorescent images containing at least three APP+ axonal swellings were taken from two sections per micro pig (eight total images). Image acquisition settings were held constant for all animals. The background was subtracted, and images were thresholded in FIJI (NIH, Bethesda, MD). The surface area and swelling roundness were assessed using the particle analysis function in FIJI and averaged for each treatment group.

### Quantification of GAP43 Expression in Injured Axonal Swellings

Following the assessment of injured axonal swelling morphology, sections were labeled with rabbit anti-GAP43 conjugated to Alexa fluorophore 488 (cat. #ab196324, 1:150, Abcam, Cambridge, MA) and remounted on superfrost slides using vectashield hardset mounting media, producing tissue doubled-labeled for both APP and GAP-43. The intensity/expression of GAP-43 within the axonal swelling in vehicle- *vs*. LEV-treated micro pigs was assessed on two systematically random sections per animal. Four images per section (eight total images) containing at least three APP+ axonal swellings were taken at × 10 magnification using a Nikon Eclipse 800 microscope equipped with an Olympus DP71 camera. The image acquisition settings were held constant for all animals, and pixel saturation was avoided. The integrated density of GAP-43 within each APP+ axonal swelling within the thalamus was assessed using FIJI image analysis software.

### Statistical Analysis

Exploratory analysis was carried out to determine the distribution of the data. The normally distributed data were analyzed *via* one-way analysis of variance (ANOVA) or paired *t*-tests. Non-parametric data were analyzed using Mann–Whitney *U* test in cases of unpaired observations or Wilcoxon signed-rank test in cases of related samples. A multilevel model was conducted in SAS PROC MIXED (version 9.4, SAS Institute, Inc., Cary, NC) to examine whether the LEV and saline groups showed different patterns of circulating GFAP or UCH-L1 across time points. Multilevel models were selected to handle the nested design with repeated measures. The variables included in the initial model were group (LEV and saline), time, and their interaction term. Statistical significance was set at a *p*-value < 0.05. Data are presented as mean ± SEM or median (interquartile range) as appropriate.

## Results

### Physiologic Parameters

To obviate potential confounds due to systemic differences between the vehicle- and LEV-treated groups, systemic physiology was monitored closely before and for 6 h after injury. Weight, injury intensity level, and duration of injury were all assessed prior to randomized therapeutic intervention to verify adherence to *a priori* exclusion criteria. Blood gas analysis for PaO_2_, PaCO_2_, pH, and hemoglobin O_2_ concentration was evaluated every hour throughout the experiment. Body temperature, heart rate, and blood pressure were all automatically recorded every 20 s. All physiology data were averaged for the entire pre- and post-injury monitoring period. As displayed in Table 1, all physiological parameters remained within normal range. The post-injury heart rate and PaO_2_ were significantly reduced following injury as compared to pre-injury measurements (heart rate: *F*_1,12_ = 3.786, *p* = 0.002; PaO_2_: *F*_1,12_ = 3.413, *p* = 0.005). Hemoglobin oxygen saturation following injury was also slightly higher in the vehicle group compared to the pre-injury LEV group (*F*_1,12_ = 4.938, *p* = 0.046); however, all physiological readouts were within normal ranges in all groups throughout the experiment. The PaO_2_ was higher than typically reported in all animals at all time points due to our use of 100% O_2_ to ventilate the animals. While these values are high, 100% O_2_ is routinely used in the clinical treatment of moderate and severe TBI, particularly early after injury, and is also often used in brain tissue oxygen-directed therapy (31). There did not appear to be any effects of LEV treatment on acute systemic physiology or blood gases.

### Biomarker Assessment

**Figure 1:**
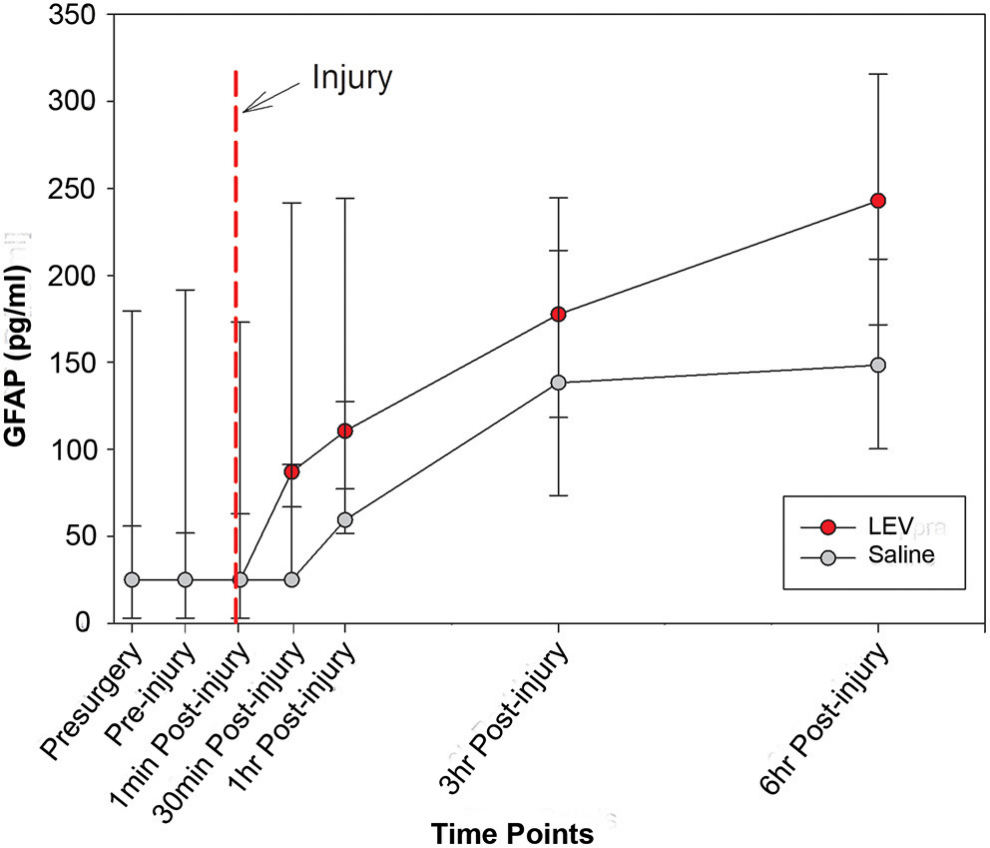
Line graph depicting the median concentration of glial fibrillary acidic protein (GFAP) in the serum of pigs treated with saline (gray) or levetiracetam (LEV; red) at various pre- and post-injury time points (saline, *n* = 7; LEV, *n* = 7). The red dashed line indicates the time of injury. Note that there were no significant differences in GFAP serum levels between vehicle- and LEV-treated pigs at any given time point. However, both groups displayed a significant increase in GFAP serum levels from baseline to post-injury, and the LEV-treated pigs had an even greater magnitude increase of GFAP serum levels post-injury compared to saline-treated pigs. Error bars represent interquartile range.

The median concentrations of GFAP and UCH-L1, stratified according to group and time, are shown in Table 2. The levels of UCH-L1 did not change over time in either treatment group. Although the pre-injury GFAP serum biomarker levels did not differ by group, a multilevel analysis shows that 37% of the variance in GFAP levels at baseline exists between animals. In addition, GFAP in blood significantly increased in both groups from baseline to post-injury assessment (*F*_1,84_ = 76.91, *p* < 0.0001), with no variation between animals, thus confirming the severity and the consistency of the injury received (Figure 1). However, in the LEV-treated group, the magnitude of GFAP increase in serum was greater than in the vehicle-treated group (*F*_1,11_ = 5.67, *p* = 0.036).

**Table 2:** Serum biomarker concentrations (pg/ml) of vehicle- and levetiracetam-treated micro pigs prior to and throughout the 6-h post-injury period.

		**Pre-surgery**	**Pre-injury post-surgery**	**1 min post-injury**	**30 min post-injury**	**1 h post-injury**	**3 h post-injury**	**6 h post-injury**
Glial fibrillary acidic protein	Levetiracetam	25 (25–179.5)	25 (25–191.6)	25 (25–173.1)	86.94 (66.9–241.7)	110.5 (77.23–244.3)	177.6 (118.3–244.5)	242.9 (171.6–315.6)
	Vehicle	25 (3–55.84)	25 (3–51.9)	25 (3–62.83)	25 (25–91.22)	59.39 (51.7–127.4)	138.3 (73.5–214.3)	148.4 (100.3–209.4)
Ubiquitin carboxy-terminal hydrolase L1	Levetiracetam	50 (50–129.5)	50 (50–50)	50 (50–139.1)	50 (50–50)	50 (50–50)	50 (50–50)	50 (50–129)
	Vehicle	50 (50–173.8)	50 (50–50)	50 (50–152.9)	50 (50–50)	50 (50–148.7)	50 (50–50)	50 (50–109.4)

Biomarker data are given as median (interquartile range).

### Histological Outcomes

**Figure 2:**
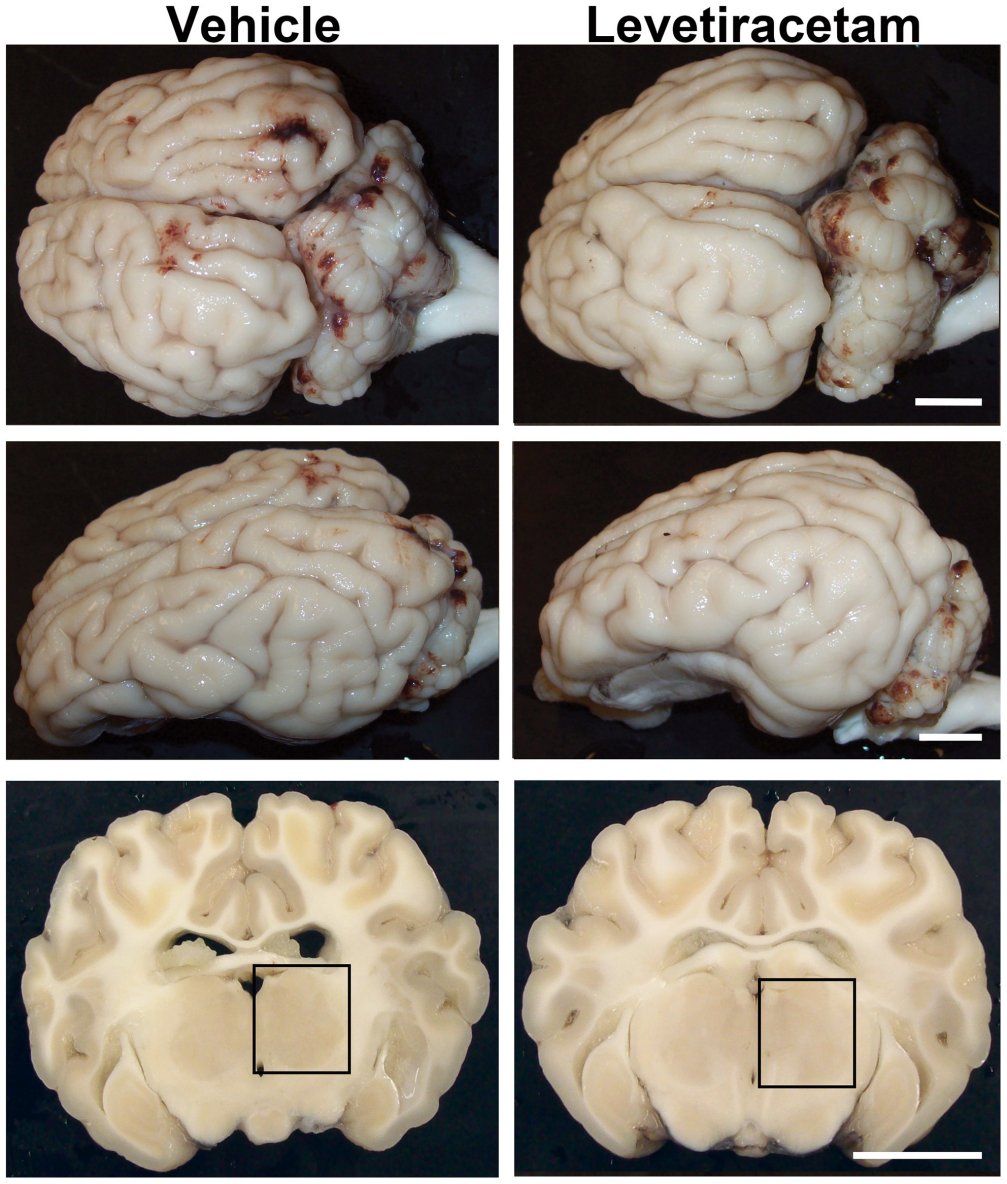
Gross brain pathology is consistent between vehicle- and levetiracetam (LEV)-treated micro pigs acutely post-central fluid percussion injury (cFPI). Representative photographs of the gross micro pig brain 6 h following cFPI and either vehicle or LEV treatment. The top panel is a dorsal view, while the middle panel is a lateral view of the whole pig brain. The lower panel represents coronal sections taken ~1–3 mm posterior to the bregma. The boxes indicate the regions of subsequent assessment of axonal injury in the thalamus (black box). Note that while the gross pathology is minimal, it appears equivalent between vehicle- and LEV-treated micro pigs. Scale bar = 10 mm.

As previously reported, cFPI is a mild TBI model, which resulted in subarachnoid bleeding dorsal to the occipital cortex and cerebellum as well as limited petechial hemorrhage without macroscopic hemorrhage within the brain parenchyma (24, 26). The degree of subarachnoid bleeding varied between animals; however, acute gross brain pathology, including hematoma, contusion, and/or mass cell death, was not present in either vehicle- or LEV-treated micro pigs, highlighting the mild nature of this injury model (Figure 2).

**Figure 3:**
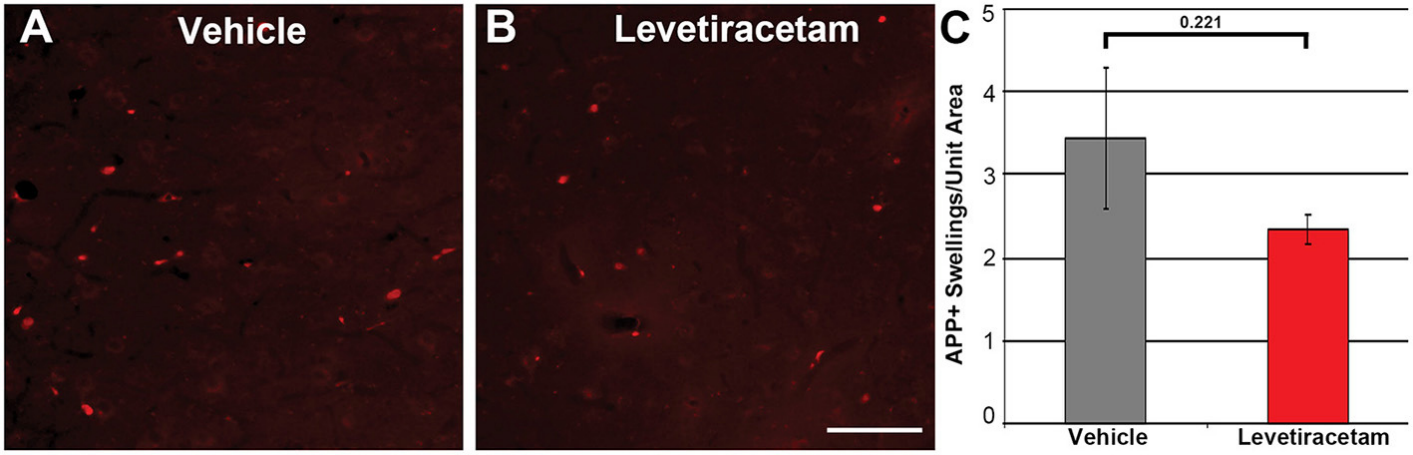
Representative micrographs of amyloid precursor protein (APP) immunofluorescence in the thalamus of pigs sustaining central fluid percussion injury (cFPI) followed by **(A)** vehicle or **(B)** levetiracetam (LEV) treatment. **(C)** Bar graph depicting the average number of APP-labeled axonal swelling/unit area of thalamic tissue at 6 h post-cFPI (vehicle, *n* = 7 pigs; LEV, *n* = 7 pigs). The number of axonal swellings at 6 h was not significantly different with LEV treatment vs. vehicle. Scale = 100 um.

**Figure 4:**
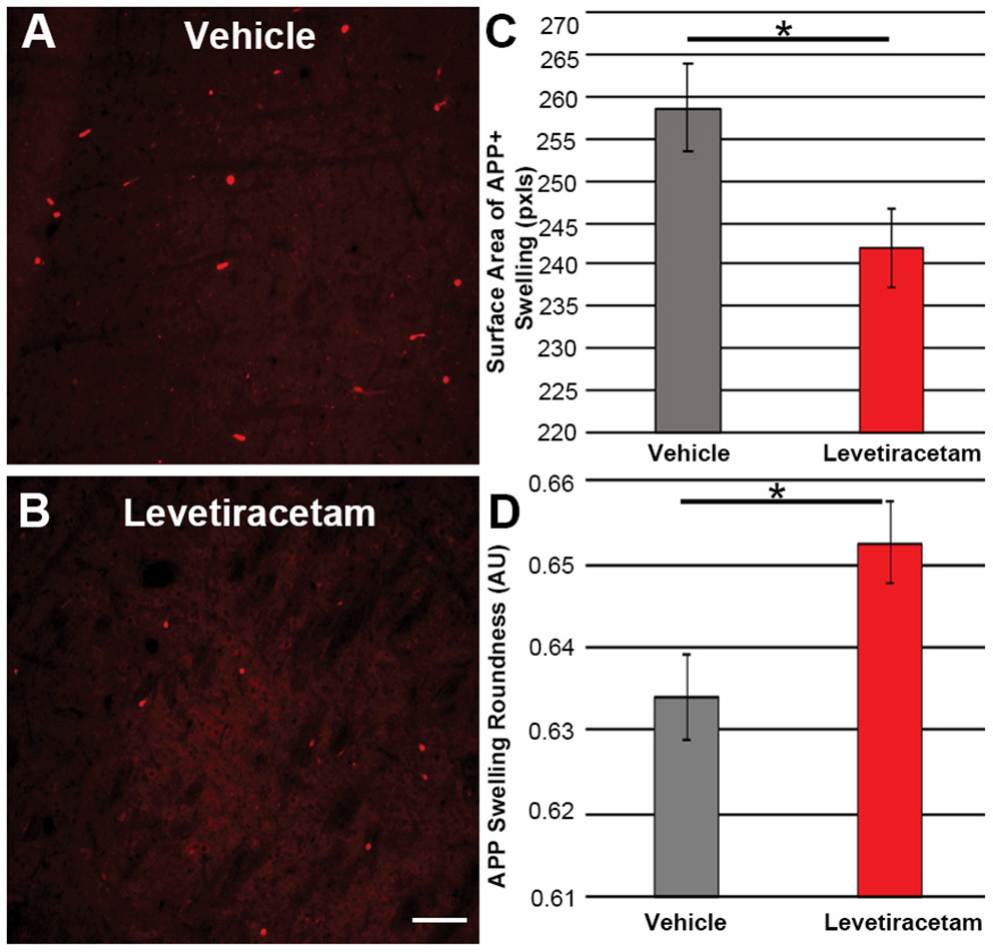
Representative micrographs of amyloid precursor protein (APP) + axonal swelling morphology in the thalamus of pigs sustaining central fluid percussion injury followed by **(A)** vehicle or **(B)** levetiracetam (LEV) treatment. The bar graphs depict the **(C)** average surface area of axonal swellings at 6 h and the **(D)** roundness of individual axonal swellings at 6 h (vehicle, *n* = 1,474 swellings from seven pigs; LEV, *n* = 1,421 swellings from seven pigs). Note the significant alteration in the morphological properties of the APP+ axonal swellings, including the reduced swelling area and the increased axonal swelling roundness observed. One-way ANOVA; error bars represent SEM. *p < 0.005. Scale = 100 um.

The thalamus was chosen as the region of interest for histological assessment based on the consistent involvement of the thalamic domain in TBI as well as our previous studies demonstrating thalamic involvement in our pig model of cFPI (24, 26, 30). While we previously observed multiple loci that demonstrated axonal injury, thalamic DAI was found to be the most consistent and quantifiable in micro pig following cFPI (24). Axonal damage within the micro pig thalamus at 6 h following cFPI and either vehicle or LEV treatment was therefore analyzed. As illustrated in Figure 3, there was no difference in the overall numbers of APP+ injured axons following LEV treatment (*p* = 0.221). Thalamic axonal swellings, however, were significantly smaller in size (*F*_1,2893_ = 5.628, *p* = 0.018; Figures 4A–C) and had a more rounded morphology (*F*_1,2893_ = 7.185, *p* = 0.007; Figure 4D) at 6 h following cFPI and LEV treatment compared to the vehicle control group.

**Figure 5:**
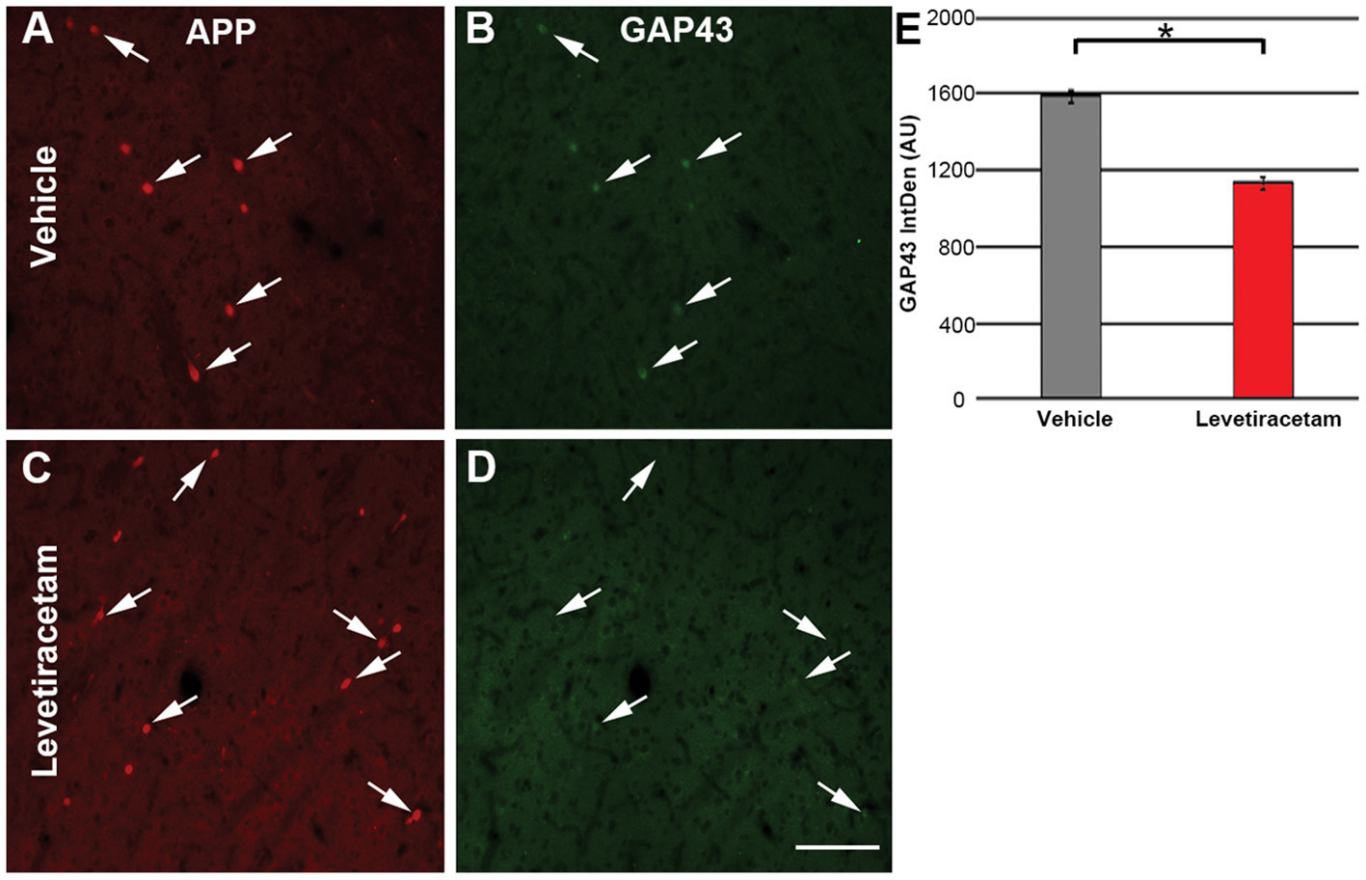
Representative micrographs of **(A,C)** amyloid precursor protein and correlative **(B,D)** GAP43 labeling in the thalamus of micro pigs 6 h following central fluid percussion injury and treated with either **(A,B**) vehicle or **(C,D)** levetiracetam (LEV). **(E)** Bar graph depicting the average integrated density of GAP43 labeling in regions of axonal swelling. Interestingly, the intensity of GAP43 was significantly lower in the LEV group as compared to the vehicle-treated pigs (vehicle, *n* = 939 swellings from seven animals; LEV, *n* = 1,007 swellings from seven animals). One-way ANOVA; error bars represent SEM. *p < 0.005. Scale = 100 um.

Since LEV treatment was associated with morphological alteration of acute thalamic DAI and LEV has been shown to alter GAP43 expression (18, 20), a common marker of neurite outgrowth (21, 32, 33), we evaluated the intensity of GAP43 labeling in the axonal swellings of pigs treated with vehicle or LEV. The expression/intensity of GAP43 in APP + thalamic axonal swellings at 6 h following cFPI and LEV treatment was significantly reduced compared to axonal swellings within the thalamus of vehicle-treated injured micro pigs (*F*_1,1944_ = 96.648, *p* = 2.7 × 10^22^; Figure 5).

## Discussion

To date, LEV has proven to be the most promising drug tested by OBTT, demonstrating therapeutic efficacy across multiple outcomes in rodent models, and was therefore advanced to testing in our micro pig model of mild diffuse TBI (22). Our approach to dosing of LEV in micro pigs was based on several lines of evidence. First, in studies of LEV across models in OBTT, doses of either 54 or 170 mg/kg were equally effective, given as a single infusion at 15 min after TBI (34, 35). LEV is unusual among drugs in that translation from rodent to large animal or human does not follow conventional scaling based on body surface area (36). It is rather better modeled based on body weight (kg) allometric scaling. In our prior rodent studies, the 54-mg/kg dose was based on the work of Wang et al. (37) using the CCI model in mice. Using a mg/kg conversion, that dose also corresponds to the conventional human dose of 3,000 mg per day (divided into three doses). The 170-mg/kg dose represented the highest dose in rats that maximally inhibited kindling of seizures without side effects based on Klitgaard et al. (38). In the absence of a direct comparison of plasma or tissue levels of LEV and/or evidence of target engagement, the use of 170-mg/kg dose in the micro pig was thus selected to maximize the chance of detecting a therapeutic signal. As the majority of the cognitive benefit of LEV treatment was seen in the LFPI model, the only OBTT rodent model with a diffuse pathology component (15, 24), the use of a diffuse injury model in micro pigs was considered to be appropriate.

Recently, there has been increased recognition of the need to raise the bar on the standard for preclinical research which demands rigor and robustness in designing, standardizing, and validating animal models as well as demonstration of reproducibility prior to clinical translation (39). Pigs possess cytoarchitecture, inflammatory responses, gene expression, and metabolic rates similar to that of humans and therefore represent an ideal higher-order gyrencephalic animal model to bridge the gap between rodent studies and human translation (24, 26). As characterized previously, our micro pig model of cFPI yields some subarachnoid bleeding and limited petechial hemorrhaging; however, it does not produce contusion, hematoma formation, or square-wave tissue damage, all of which were consistent with a milder form of TBI (24, 26). Importantly, the GFAP patterns that emerged in this investigation were also consistent with a lower level of severity while confirming the reproducibility of the injury, thereby providing evidence that our model behaves in a reliable and predictable fashion. These results are in line with and extend our previous work (13, 25), showing that GFAP enables the reliable, specific, objective, and measurable assessment and characterization of TBI models while providing a robust framework for its use as a tool in different species. The UCH-L1 serum biomarker levels, however, may not be as reliable in this pig models of diffuse TBI as was highlighted in our previous study (25). The addition of a pathobiologically diverse set of biomarkers, including their exosomal component, may substantially improve the current approach, enabling us to reflect and gauge the response to therapy more effectively (38–41). However, blood-based brain injury biomarker research in large animal models is in its infancy, and the majority of the assays have been not specifically tested and validated in pigs, thereby limiting their reliability and application and requiring further study (42).

In accordance with our previous studies, aside from the single vehicle-treated animal that was excluded from our assessments, no focal brain damage was identified at 6 h following cFPI in either vehicle- or LEV-treated animals, indicating that LEV does not adversely affect the gross pathological progression of cFPI or induce massive cell death. Additionally, there were no differences in systemic physiology between vehicle and LEV treatments aside from a slight, but significant, change in hemoglobin oxygen saturation. Importantly, all physiological readings, including hemoglobin O_2_%, remained well-within normal limits, signifying that LEV treatment did not negatively affect systemic physiology in micro pigs up to 6 h post-cFPI.

One of the histological hallmarks of TBI is diffuse axonal injury (DAI), in which physical forces during trauma precipitate axonal injury that progresses to disconnection, leaving a proximal axonal segment that remains connected to the neuronal soma and a distal axonal segment that undergoes Wallerian degeneration (43–46). The proximal axonal segment ends in a swelling of pooled organelles and proteins, which is commonly visualized using antibodies targeting the ubiquitously expressed and anterogradely transported protein, APP. As micro pig cFPI, as used in our studies, produced a mild diffuse TBI, histological metrics involved the assessment of APP+ injured axonal swellings as opposed to lesion/contusion volume, which was done in our previous rodent studies.

While we previously observed multiple loci that demonstrated APP+ axonal injury, thalamic DAI was found to be most consistent and quantifiable in the micro pig following cFPI and, therefore, was assessed in the current study (24). There were no differences in the number of APP+ axonal swellings within the micro pig thalamus at 6 h following cFPI between vehicle *vs*. LEV treatment. However, there were significant alterations in the morphological properties of the APP+ axonal swellings, including reduced swelling area and increased swelling roundness. Proximal axonal swellings that label with APP are sites of potential neurite outgrowth (47–49). The elongated or torpedo-shaped morphology of axonal swellings is indicative of potential outgrowth (47, 48, 50). Previous studies have demonstrated evidence of potential axonal outgrowth following TBI (47, 48, 51). Our current findings indicate that LEV treatment reduces potential morphological alterations linked to neurite outgrowth at 6 h following cFPI in the micro pig.

Studies have also shown increases in GAP43, a common marker of neurite outgrowth, following TBI (49, 52–54). Additionally, administration of LEV following focal brain injury demonstrated increased GAP43 expression (20). Strikingly, the current study demonstrated a significant decrease in GAP43 expression in the APP+ axonal swellings of micro pigs following cFPI and LEV treatment compared to vehicle-treated micro pigs. These findings are surprising as LEV treatment *in vitro* is associated with enhanced neurite outgrowth and increases in overall GAP43 expression *via* a mechanism involving the binding of SV2a (18). However, in these studies, GAP43 expression appears to be upregulated at more subacute time points (at least 1 day post-injury) as compared to the 6-h post-cFPI time point investigated in the current study (55). The observed implications of LEV-associated reductions in outgrowth of injured axons at 6 h post-injury in the current study could be indicative of a detrimental effect, which is also suggested by the greater post-injury GFAP increases in LEV-treated animals. On the other hand, these findings also fit the hypothesis that LEV exerts a beneficial effect by reducing post-traumatic neural reactive axonal sprouting (56). In support of this possibility, treatment with antiepileptic drugs, such as LEV, is associated with a reduced risk of developing post-traumatic epilepsy up to 9 years following TBI (6). A study of lithium–pilocarpine-induced epilepsy in rats found that treatment with LEV reduced the GAP43 levels in a dose-related fashion, theorizing that reduction in axonal sprouting may be a mechanism for LEV's antiepileptogenic functions (57). Several lines of evidence have shown associations between episodes of excessive neural activity and aberrant axonal and dendritic sprouting and maladaptive plasticity, which contribute to negative cognitive and behavioral outcomes (47, 58–61). The observed alterations in GAP43 and axonal swelling morphology indicative of reductions in neurite outgrowth could represent the beginning movements toward epileptogenesis after TBI. These studies may help identify a unique pathology linking TBI to progressive epilepsy and could provide an even stronger rationale for the use of LEV following TBI. Furthermore, it is possible that LEV induces a “biphasic response,” characterized by an early inhibitory effect followed by delayed facilitatory effects on functional recovery and axonal regeneration. Future studies with extended temporal profile and sampling will be required to address this dilemma.

The current study showed more muted differences between LEV and vehicle as compared to those indicated from our previous OBTT rodent studies (22) and the work of Wang et al. (37) in a closed head injury model that produced neuronal death in mice, largely on which the OBTT pursuit of LEV was based. The micro pigs did, however, receive a different subtype of injury as compared to the previous rodent studies. Specifically, micro pig cFPI is a mild diffuse TBI, whereas the LFPI, CCI, and PBBI models used for our rodent studies are moderate to severe TBI models with a prominent focal lesion. The rodent OBTT model most similar to the micro pig cFPI would be the LFPI rodent model; however, even this model has a contusion component not recapitulated in the pig cFPI model (15, 24, 26). Consideration of the extremely complex heterogeneity of clinical TBI that cannot be adequately mimicked by a single animal model is triggering a transformative approach and an unprecedented evolution in understanding specific pathological mechanisms and injuries and relevant phenotypes. Multimodal multi-marker bio-signatures must be developed to improve therapeutic decision-making beyond current practice standards and open up the possibility of new adaptive trial designs which facilitate patients' access to drugs with promising activity for their own specific injury.

The time point for terminal assessment of 6 h in the micro pig study was rather acute compared to our previous rat assessments where neuropathology was assessed at 21 days. Indeed higher GFAP levels were seen with LEV treatment in the micro pig beginning at the remarkably early post-injury time of 30 min. In addition, the rodent GFAP serum biomarker levels were only seen to decrease below vehicle in the CCI model at 1 day post-injury treated with the high LEV dose (22). Indeed examination of the serum GFAP levels at 4 h after TBI in rats across all models as shown in prior studies by OBTT revealed no reductions in LEV vs. vehicle groups. In addition, the reduction in GFAP by LEV *vs*. vehicle at 24 h after injury was seen only in CCI and PBBI—models that produce much more neuronal death and focal injury than LFPI. These findings suggest potential model dependence for utility of GFAP in pharmacodynamics response monitoring of therapies. Nevertheless, the mechanistic underpinning of the increase in serum GFAP early after cFPI in these studies remains to be determined. Additionally, in our previous work, cognitive testing of injured rodents occurred 2 to 3 weeks following LFPI, CCI, and PBBI and was a key metric upon which LEV showed the most promise in OBTT's previous studies (22). However, due to the acute terminal time point for the current study, cognitive and motor functions were not assessed in the micro pig, and therefore LEV's potential effects on cognition in a higher-order animal remain unknown and will be addressed in future studies.

Baseline biomarker levels also varied between animals. This may be a result of animal characteristics or analytical aspects/issues; however, further investigation is warranted to fully explore the high degree of animal-to-animal variability. Of note is the fact that this contrasts the low level of baseline variability seen across the rat models (13). We also recognize that there were differences between the anesthetic approaches taken in the rat vs. the micro pig studies across OBTT. Although isoflurane was used as a maintenance anesthetic in all models across OBTT, tiletamine, a component of telazol, a known non-competitive NMDA receptor antagonist and pentobarbital, also an anti-excitotoxic agent, was used for induction in the micro pigs, which could impact the therapeutic efficacy of LEV and potentially yield complex interactions with LEV given its mechanism(s) of action (62, 63). Indeed in some studies tiletamine has shown paradoxical pro-convulsant actions (64). Other modeling differences could also be involved.

Finally, while the metabolism between rodents (~7 × that of humans) and micro pigs is different, a later time point of 1 day, 1 week, or even 1 month post-injury might be more telling in terms of potential drug effects. Indeed Bramlett et al. (65) reported a delayed increase in axonal injury in the thalamus at 7–30 days after LFPI in rats, further supporting the importance of examining later time points in our micro pig model. However, this comes with the caveat that longer survival for large animals requires much more facilities and technical and staffing support, therefore much more financial investment, compared to rodent studies. Due to the high degree of variability and need to assess higher-order animals at much more chronic post-injury time points, using rodents and other lower-order animal models for initial assessments and/or screening of drug efficacy prior to moving trials to a high-order gyrencephalic animal is an economical solution (66, 67). This schema is what OBTT has strived for to enhance efficiency while investigating clinically relevant therapeutics with the goal of expeditious movement to randomized clinical trials, either across or within injury endophenotypes (14). The current findings illustrate the need for additional studies to elucidate the effects of LEV treatment on potential maladaptive neuroplasticity, neuroinflammatory pathways, and neurite outgrowth over longer time points following diffuse TBI.

## Data Availability Statement

The raw data supporting the conclusions of this article will be made available by the authors, without undue reservation.

## Ethics Statement

The animal study was reviewed and approved by Institutional Animal Care and Use Committee, Virginia Commonwealth University.

## Author Contributions

AL designed and coordinated the study, designed and carried out the microscopic analyses, and wrote the manuscript. SM analyzed the biomarker data and wrote the manuscript. JP participated in the micro pig surgeries and the ultrastructural analyses, conceived the study and participated in its design, and wrote the manuscript. SW participated in the micro pig surgeries and the tissue processing and design for/of the microscopy studies. KG collected and prepared the serum samples and brain tissue and participated in the design and analysis of the microscopy studies. KW and RH coordinated and analyzed the serum biomarkers. PK conceived, designed, and coordinated the study. All authors contributed to the article and approved the submitted version.

## Conflict of Interest

RH owns stock and is an officer of Banyan Biomarkers, Inc., receives salary from and owns stock in Banyan Biomarkers Inc. KW is a former employee of Banyan Biomarkers, Inc., and owns stock in Banyan Biomarkers, Inc. RH and KW also receive royalties from licensing fees of the serum biomarker used in this study and, as such, may benefit financially as a result of the outcomes of this research or work reported in this publication. The remaining authors declare that the research was conducted in the absence of any commercial or financial relationships that could be construed as a potential conflict of interest.
